# Attitude and Knowledge of Indian Emergency Care Residents towards Use of Proton Pump Inhibitors

**DOI:** 10.1155/2014/968430

**Published:** 2014-11-19

**Authors:** Biswa Mohan Padhy, Hemant Singh Bhadauria, Yogendra Kumar Gupta

**Affiliations:** ^1^Department of Pharmacology, All India Institute of Medical Sciences, Sijua, Bhubaneswar, Odisha 751019, India; ^2^Department of Pharmacology, All India Institute of Medical Sciences, Ansari Nagar, New Delhi, Delhi 110029, India

## Abstract

*Objective*. Several studies carried out in developed countries have reported disproportionately high usage of acid suppressive drugs, especially proton pump inhibitors (PPIs). However, systematic assessment of attitude and practices of health care providers towards the use of these drugs in developing countries is lacking. In this study, we assessed the knowledge, attitude, and preferences of resident doctors posted in the emergency department of a tertiary care hospital in North India, towards the use of PPIs. *Methods*. A questionnaire based survey was carried out. *Results*. Fifty resident doctors responded to the questionnaire. Thirty-six percent reported prescribing acid suppressive drugs for majority of their patients, while 12% prescribed them to almost all patients they attended. Acute gastritis was the most common indication for prescribing PPI/H_2_ blockers (50%). The majority of respondents (92%) regarded PPIs as their first choice in acid suppressive agents and 58% administered it through intravenous route. Knowledge about PPI related adverse effects was low. *Conclusions*. Emergency care residents in India also tend to overuse PPIs in a manner similar to their counterparts in developed countries. Specific measures may be helpful in preventing such practices.

## 1. Introduction

The proton pump inhibitors (PPIs) are the most widely prescribed class of drugs used to suppress gastric acid secretion. They are used to treat peptic ulcer disease (PUD), gastroesophageal reflux disease (GERD), erosive esophagitis, Zollinger-Ellison syndrome, Barrett's esophagus, and upper gastrointestinal bleeding [1]. PPIs have been proven to be superior in the treatment and symptomatic remission of nonerosive reflux disease and erosive esophagitis compared to H_2_ antagonists [2]. They are also used for stress ulcer prophylaxis (SUP) and as gastroprotective agents along with NSAIDs. However, due to poor implementation and low compliance with the available guidelines, PPIs are being used indiscriminately in both the intensive and nonintensive care settings [2, 3].

In 2006, the global expenditure on PPIs was 7 billion USD, whereas between April 2013 and March 2014, the PPI esomeprazole (Nexium) was the third best-selling drug in the USA with 19.3 million prescriptions and revenue of nearly 6.3 billion USD [2, 4]. The dramatic increase in PPI prescribing patterns over the past several years has raised key questions relating to their appropriate utilization [5]. There are concerns that acid suppressive therapy, predominantly with PPIs, is overutilized for the treatment of GERD and SUP leading to significant yet controllable cost expenditure [2]. It has been estimated that between 25% and 70% of patients are prescribed PPIs without appropriate indication and consequently two billion USD each year is spent unnecessarily on these medications [6]. In addition, concerns have been raised related to the inappropriate use of the intravenous (IV) route of administration and to a lesser extent incorrect doses and length of therapy. Furthermore, often patients are inappropriately discharged on PPIs which could potentially increase the risk of pneumonia and* Clostridium difficile* associated disease and metabolic interactions with several other drugs [5].

Many drug utilization studies have identified overutilization of PPIs at various centres, especially in the developed countries. However, there is need to have systematic assessment of perceptions and practices of health care providers towards the use of this class of drug. This is of special relevance to developing countries, particularly India, where health resources are often limited and where only 15% of the population is covered by health insurance [7]. In view of this, we carried out a questionnaire based study to assess the knowledge, attitude, and preferences of resident doctors posted in the emergency department of a tertiary care hospital in North India, towards the use of gastric acid suppressants (especially PPIs). To our knowledge, this is the first survey on the use of gastric acid suppressants in an emergency care setup from India.

## 2. Materials and Methods

### 2.1. Study Design and Population

The study was carried out using a two-page questionnaire, developed to assess the knowledge, attitude, and preferences of resident doctors posted in the emergency medicine department of All India Institute of Medical Sciences, New Delhi, towards the use of acid suppressive drugs, particularly PPIs. Written informed consent was obtained from the participants prior to responding to the questionnaire. Formal sample size calculation was not carried out, but a target of nearly 50 respondents had been planned, based on convenience sample of the average number of residents posted in the emergency department on a monthly basis. Officially, resident doctors are categorized as junior residents (those who have completed graduation-MBBS and have undergone one year internship training but have not yet obtained a postgraduate degree, M.D./M.S.) or as senior residents (those who have also obtained a specialist postgraduate degree, M.D./M.S. and have at least three years of work experience in the concerned subject). The questionnaire was developed after review of previously published studies conducted in other clinical settings [8–10]. The first part of the questionnaire consisted of questions designed to elicit demographic information (name (optional), age, sex, qualifying medical degree, speciality, and length of posting in emergency department). The second part consisted of questions regarding average number of patient encounters, requirement of IV medications, and time available to consider clinical management of cases. In addition, there were questions regarding choice of acid suppressive drug, preferred route of administration, duration of treatment, and knowledge about adverse drug reactions and potential risks with use of PPI. There was the option for multiple answers for questions on adverse drug reactions encountered and potential risks associated with PPI. The questionnaire had been piloted previously in a group of 20 resident doctors from the departments of surgery and medicine and tested for validity and reliability (Cronbach's *α* = 0.76) before administration to emergency department residents. The study was carried out after obtaining approval from the institute ethics committee.

### 2.2. Statistical Analysis

Data was entered into Microsoft Excel (MS Office 2007) and statistical analyses were conducted using SPSS ver. 17.0 (SPSS, Chicago, IL). Multiple regression analysis was used to assess the effect of age, sex, duration of posting, and designation on the responses. The *P* value <0.05 was considered as significant. Epicalc 2000 software was used to calculate 95% confidence intervals [11].

## 3. Results

### 3.1. Demographic Attributes and Particulars of the Respondents

Fifty resident doctors participated in the study. The majority of respondents were male (74%) and were of average age of 27.3 ± 2.3 years. While a number of the respondents had basic medical qualification MBBS (74%), only 20% had additional postgraduation (M.D. or M.S.) qualifications. Most of the respondents were from the department of emergency medicine having average work experience of less than 6 months (Table 1).

### 3.2. Specific Prescribing Practices

Half of the respondents reported 10–20 patient encounters every day, 18% reported 21–30 patient encounters per day, and 32% reported attending to more than 30 patients daily. Majority of respondents (80%) stated that they did not get adequate time to critically consider the drug therapy appropriate for the clinical condition of the patient. Forty-eight percent of the residents felt that up to 60–89% of the patients presenting to the emergency require some sort of medications including IV fluids (data not shown in Table 2). All the responding resident doctors stated that they prescribed antiulcer drugs to their patients. Most reported prescribing acid suppressive drugs (PPI and H_2_ blockers) for their patients, with 12% of them prescribing these acid suppressive drugs to almost all the patients they attended (90–100%).

Half of the respondents stated that acute gastritis was the most common indication for prescribing PPI or H_2_ blockers; 22% used them for prophylaxis against stress ulcers. Many residents did not have enough time to read the package insert of the acid suppressive agents they prescribe (Table 2). Regression analysis revealed that the demographic profile of the respondents including age [coefficient (B) = −0.116, 95% CI (−0.228, 0.142), *P* = 0.639]; sex [coefficient (B) = 0.191, 95% CI (−0.342, 1.044), *P* = 0.311]; and the duration of posting in the emergency department [coefficient (B) = −0.085, 95% CI (−0.061, 0.040), *P* = 0.673] had no statistically significant influence on the prescription of acid suppressive drugs. However, there was a significant negative correlation of the resident's age with not finding enough time to read the package inserts [coefficient (B) = −0.268, 95% CI (−0.467, −0.069), *P* = 0.01].

### 3.3. Practices and Knowledge regarding PPI

The majority of the respondents (92%) regarded PPIs as their first choice as an acid suppressive agent. Fifty-eight percent of the residents preferred the intravenous route (IV) for administration of PPI. The duration of treatment of less than 1 week was preferred by many residents. Most residents (82%) replied that the majority of the patients procured the drug from the hospital free of cost. Only three residents (6%) stated that they had encountered an adverse event with PPI with each stating hypersensitivity, nausea, vomiting, and diarrhoea as the presenting event. When knowledge about possible adverse events with PPI was solicited, a total of 44 answers were obtained from 30 (60%) residents who chose to respond to this question. Twenty-two (44%) of them gave a single answer while the rest responded with more than one answer. From the responses obtained, it seemed that only 10% of the residents included in the study were aware of increased risk of community acquired pneumonia, 22% of increased risk of* Clostridium difficile* infection, 12% of increased risk of hip fractures, and 44% of the decrease in absorption of Vit. B_12_ (Tables 2 and 3).

## 4. Discussion

In this study, 32% of the respondents claiming to attend to more than 30 patients per day reiterate the underlying stress and time constraints faced by residents in the emergency setup. Consequently, the majority of the physicians do not get adequate time to consider the drugs most appropriate for the patient's clinical condition. In a typical emergency department, patients are treated in an environment characterized by high volume, high acuity, clinical uncertainty, multiple handoffs, and staff shortages. Therefore, there is a tendency to use medications and IV fluids for symptomatic relief and to assuage the anxiety of the patients and their relatives [12, 13]. In the present study, this behaviour was found to be widespread without bearing any correlation with demographic profile or duration of posting in the emergency department. However, the study setting was a tertiary care hospital where patients are referred from different states and often arrive in precarious condition, mandating parenteral medications and fluids [13, 14]. Nevertheless, such practices increase the chances of medication errors, putting tremendous pressure on the health care system and driving up costs of hospitalization and management of adverse drug reactions (a third of which are due to medication errors) [14].

Our survey revealed that the majority of the respondents' prescribed acid suppressive drugs to a high proportion of their patients. Half of the respondents stated acute gastritis as the most common indication for prescribing acid suppressive drugs and 22% prescribed them for prophylaxis of stress ulcers and 20% along with NSAIDs. Considering that no burn or trauma cases are registered in the department of emergency medicine as there is a separate unit for such cases, it seems that PPIs were inappropriately used for SUP. Evidence of superiority of PPIs over H_2_ blockers is much less compelling in patients who have functional dyspepsia or who have gastroesophageal symptoms without evidence of a lesion. Therefore, most cases diagnosed as acute gastritis in our emergency department could possibly have been managed by H_2_ blockers, unless specific indications for PPIs existed. The widespread use of acid suppressive therapy for stress-ulcer prophylaxis in general medical settings has been recognized, especially among patients cared for by medical residents [15]. Despite the presence of guidelines that delineate the limited populations that derive benefit from stress ulcer prophylaxis (SUP), an estimated 22% to 54% of hospitalized patients receive these “prophylactic” gastroprotective agents, mostly PPIs [2]. Patients with chronic conditions requiring NSAIDs should receive either a coxib or concomitant therapy with misoprostol or a PPI if GI risk factors are present. However, such patients are often prescribed gastroprotective drugs empirically, without proper evaluation for GI risk factors [16, 17]. Interestingly, the inappropriate use of PPIs and H_2_ blockers as prophylactic agents in Indian patients on low dose aspirin has also been reported [18].

In our study, most residents considered PPIs as their first choice in acid suppressive drugs. This was possibly because PPIs are considered highly efficacious and potentially devoid of adverse effects. This trend of preferring PPIs over H_2_ blockers is prevalent globally. In 2007, esomeprazole, lansoprazole, and pantoprazole were the fourth, eighth, and thirteenth leading prescription drugs dispensed in the United States, with 26.4, 20.4, and 16.1 million prescriptions, respectively. Comparatively, ranitidine and famotidine were ranked 47th and 120th with 13 and 3 million prescriptions, respectively [1]. Furthermore, in our survey more than half of the residents preferred the IV route over oral route for administering PPIs. Intravenous PPI is indicated in the treatment of upper GI bleeding caused (UGIB) by PUD associated with high risk stigmata and in patients requiring a PPI but who cannot take medications orally [19]. Many European and North American studies have suggested widespread and inappropriate use of IV PPIs in various medical institutions [20]. In a survey evaluating residents' use and understanding of IV PPI administration, 36% preferred IV pantoprazole for conditions in which there was no clear clinical benefit [21]. Emergency physicians often encounter patients with UGIBs who may experience significant delays before undergoing endoscopy. However, only a small proportion of all patients with UGIBs have high risk lesions on endoscopy that would mandate the use of IV PPIs [22, 23]. Recent studies have also revealed interesting trends associated with use of IV PPI such as prescriptions written by junior doctors, in female patients, in the elderly, in admission to surgical ward, and in prescriptions written during the evening and night when staff supervision is low [24, 25]. A multifaceted approach including physician/pharmacist education, IV PPI ordering templates, and guidelines may lead to more appropriate use of IV PPIs [20].

The cost implications of inappropriate prescribing of PPIs are also significant. In the United States generic H_2_-blockers cost less than 20 USD per month as compared to PPIs which cost 120 USD per month and such cost differential is also observed in India [1]. As the relative proportion of residents preferring PPIs over H_2_ blockers was very high in our study, treatment cost increments would be inevitable. Most respondents were aware that the cost of PPI was not borne by the patient but by the hospital and yet preferred PPIs over other gastroprotectants possibly due to unrestricted access to PPIs [26]. Therefore, proven strategies such as step-down, step-up and on-demand therapy with PPIs should also be adopted in our emergency department to improve the cost-effectiveness of PPI based treatment [1].

While 38% of the residents did not have enough time to read the package inserts, 26% had never actually thought about reading it. In the hectic backdrop of the emergency department, the package insert is often a ready source of drug information available to the physician. It lists the therapeutic indications, method of administration, contraindications, special warnings and precautions, and other important clinical pharmacology related information. Although inadequate at times, the nature and quality of prescribing information in the package inserts accompanying drug products in India have been steadily improving over time and may act as useful reference [27]. However, not reading the package inserts of acid suppressants that belong to relatively safe category of drugs should not be considered as contrary to good prescribing practice.

In our survey, very few respondents had encountered an adverse drug reaction with PPIs in the emergency. Although the rate of adverse drug reactions with PPIs has been shown to be low, the possibility of occurrence of life threatening anaphylactic reactions should always be considered. The risk of occurrence of such reactions is also present with other drugs used in emergency care, especially those which are administered intravenously. Many recent studies have brought to attention infrequent but serious multisystem adverse effects of PPI therapy such as increased incidence of* Clostridium difficile* colitis, community acquired pneumonia, acute interstitial nephritis, vitamin B_12_ deficiency, increased risk of developing hip fractures, esophageal and noncardia gastric adenocarcinoma, and sporadic duodenal G-cell tumors [28]. As PPIs are metabolized through the liver via the cytochrome P450 (2C19) pathway they have the potential of altering the plasma concentrations of coadministered medications such as phenytoin and warfarin that are metabolized by the same system [6]. In the present study, we noted the low awareness about the possible risks associated with chronic PPIs use. A recent web-based survey to assess physician's knowledge, beliefs, and behaviour surrounding the prescribing of SUP for non-ICU patients revealed that ignorance of the adverse effects of acid suppressive therapy was strongly associated with inappropriate prescribing of the agents [29]. Despite the fact that most of our respondents preferred to prescribe PPIs for short durations, awareness about the complications associated with chronic use of PPIs is imperative and can be raised by systematic educational methods. Some limitations of this study like the purely descriptive nature of the study, small sample size of respondents, exclusion of nursing staff of the emergency department, and conduct of the survey at a single government run institution should be noted. Therefore, the results of our survey may not reflect national practice patterns.

## 5. Conclusions

Our survey reveals that emergency care residents in India also tend to prescribe gastric acid suppressants, particularly PPIs in a manner similar to their counterparts from developed countries. Being efficacious suppressors of gastric acid secretion, PPIs have become highly popular antiulcer agents worldwide over the past few years. However, inappropriate use of PPIs gives rise to concerns regarding adverse effects and imposes burden of increased treatment costs on the individual as well as on the government health care system. Therefore, in developing countries like India, overprescription of PPIs should be curtailed by implementing specific guidelines for their use and promoting their rational use.

## Figures and Tables

**Table 1:** Demographic profile of the respondents. Data presented are number responded (%) except for age and duration of posting (*N* = 50).

Demographic variable	
Mean age (yr) ± SD	27.3 ± 2.28
Mean duration of posting in emergency	4.39 ± 6.5
(months) ± SD	
Male	37 (74%)
Female	13 (26%)
Designation	
Senior residents	10 (20%)
Junior residents	36 (72%)
Not specified	4 (8%)
Educational qualification	
Graduation (MBBS)	37 (74%)
Postgraduation (M.D.)	8 (16%)
Postgraduation (M.S.)	2 (4%)
Not specified	3 (6%)

**Table 2:** Emergency care residents' views on use of acid suppressing drugs (*N* = 50).

Question	Number responded	%, [95% CI]
Do you prescribe acid suppressing drugs for		
your patients?		
Yes	50	100% [91.11, 99.82]
No	0	—

What percentage of patients require acid		
suppressing drugs?		
90–100%	6	12% [4.97, 25.00]
60–89%	20	40% [26.73, 54.80]
30–59%	18	36% [23.28, 50.86]
Less than 30%	6	12% [4.97, 25.00]

Most common indication for prescribing		
acid suppressing drugs		
Acute gastritis	25	50% [35.72, 64.28]
Stress ulcer prophylaxis	11	22% [11.99, 36.33]
Along with NSAIDs	10	10% [10.50, 34.14]
Others	4	8% [2.59, 20.11]

Acid suppressing drug preferably prescribed		
PPI	46	92% [79.89, 97.41]
H_2_ receptor blocker	2	4% [0.70, 14.86]
Others	2	4% [0.70, 14.86]

Do you read the package insert of the acid		
suppressing drug before prescribing?		
Yes	9	18% [9.05, 31.92]
No, not required	9	18% [9.05, 31.92]
No, do not have time	19	38% [25.00, 52.84]
No, never thought of it	13	26% [15.08, 40.61]

Most common route of administration		
preferred by you		
Oral	21	42% [28.49, 56.73]
Parenteral (IV/IM)	29	58% [43.27, 71.51]

Duration of prescribing acid suppressing drugs		
Less than 1 week	31	62% [47.16, 75.00]
1-2 weeks	13	26% [15.08, 40.61]
2–4 weeks	4	8% [2.59, 20.11]
More than 4 weeks	2	4% [0.70, 14.86]

From where do the patients procure acid		
suppressing drugs prescribed by you?		
Free of cost by hospital	41	82% [68.08, 90.95]
Purchased by patient	9	18% [9.05, 31.92]

**Table 3:** Knowledge about various adverse effects and potential risks associated with proton pump inhibitors; ^*^questions having option for multiple answers (*N* = 50).

Question	Number responded	%, [95% CI]
Have you ever encountered any adverse event during		
the administration of PPIs in emergency?		
Yes	3	6% [1.56, 17.54]
No	47	94% [82.46, 98.44]

If yes what was/were the adverse event(s) observed^*^?		
Rash/hypersensitivity	1	2% [0.10, 12.01]
Nausea/vomiting	1	2% [0.10, 12.01]
Diarrhoea	1	2 % [0.10, 12.01]

Which of the following conditions do you think can be		
attributed to the use of PPIs^*^?		
Increased risk of community acquired pneumonia	5	10% [3.74, 22.59]
Increased risk of *Clostridium difficile* infection	11	22% [11.99, 36.33]
Increased risk of hip fractures	6	12% [4.97, 25.00]
Decrease in the absorption of Vit. B_12_	22	44% [30.27, 58.65]

## References

[1] Ramser K. L., Sprabery L. R., Hamann G. L., George C. M., Will A. (2009). Results of an intervention in an academic internal medicine clinic to continue, step-down, or discontinue proton pump inhibitor therapy related to a tennessee medicaid formulary change. *Journal of Managed Care Pharmacy*.

[2] Heidelbaugh J. J., Goldberg K. L., Inadomi J. M. (2009). Overutilization of proton pump inhibitors: a review of cost-effectiveness and risk [corrected]. *The American Journal of Gastroenterology*.

[3] Eid S. M., Boueiz A., Paranji S., Mativo C., Regina Landis B. A., Abougergi M. S. (2010). Patterns and predictors of proton pump inhibitor overuse among academic and non-academic hospitalists. *Internal Medicine*.

[4] Brooks M. Top 100 Most Prescribed, Top Selling Drugs. http://www.medscape.com/viewarticle/825053.

[5] Nasser S. C., Nassif J. G., Dimassi H. I. (2010). Clinical and cost impact of intravenous proton pump inhibitor use in non-ICU patients. *World Journal of Gastroenterology*.

[6] Mullin J. M., Gabello M., Murray L. J. (2009). Proton pump inhibitors: actions and reactions. *Drug Discovery Today*.

[7] Yip W., Mahal A. (2008). The health care systems of China and India: performance and future challenges. *Health Affairs*.

[8] Lacy B. E., Crowell M. D., Riesett R. P., Mitchell A. (2005). Age, specialty, and practice setting predict gastroesophageal reflux disease prescribing behavior. *Journal of Clinical Gastroenterology*.

[9] Lanas A., Esplugues J. V., Zapardiel J., Sobreviela E. (2009). Education-based approach to addressing non-evidence-based practice in preventing NSAID-associated gastrointestinal complications. *World Journal of Gastroenterology*.

[10] Barrison A. F., Jarboe L. A., Weinberg B. M., Nimmagadda K., Sullivan L. M., Wolfe M. M. (2001). Patterns of proton pump inhibitor use in clinical practice. *The American Journal of Medicine*.

[11] Taylor D. M., Walsham N., Taylor S. E., Wong L. (2004). Use and toxicity of complementary and alternative medicines among emergency department patients. *EMA—Emergency Medicine Australasia*.

[12] Kulstad E. B., Sikka R., Sweis R. T., Kelley K. M., Rzechula K. H. (2010). ED overcrowding is associated with an increased frequency of medication errors. *The American Journal of Emergency Medicine*.

[13] Gupta M., Malhotra S., Chandra K. K., Sharma N., Pandhi P. (2004). Utilization of parenteral anti-infective agents in the medical emergency unit of a tertiary care hospital: an observational study. *Pharmacoepidemiology and Drug Safety*.

[14] Patel K. J., Kedia M. S., Bajpai D., Mehta S. S., Kshirsagar N. A., Gogtay N. J. (2007). Evaluation of the prevalence and economic burden of adverse drug reactions presenting to the medical emergency department of a tertiary referral centre: a prospective study. *BMC Clinical Pharmacology*.

[15] Janicki T., Stewart S. (2007). Stress-ulcer prophylaxis for general medical patients: a review of the evidence. *Journal of Hospital Medicine*.

[16] Tsumura H., Tamura I., Tanaka H. (2007). Prescription of nonsteroidal anti-inflammatory drugs and co-prescribed drugs for mucosal protection: analysis of the present status based on questionnaires obtained from orthopedists in Japan. *Internal Medicine*.

[17] Lanas A., Esplugues J. V., Zapardiel J., Sobreviela E. (2009). Education-based approach to addressing non-evidence-based practice in preventing NSAID-associated gastrointestinal complications. *World Journal of Gastroenterology*.

[18] Balakrishnan S., Jhaj R. (2008). Prophylactic use of gastro-protective agents in patients on low-dose aspirin. *British Journal of Clinical Pharmacology*.

[19] Kaplan G. G., Bates D., Mcdonald D., Panaccione R., Romagnuolo J. (2005). Inappropriate use of intravenous pantoprazole: extent of the problem and successful solutions. *Clinical Gastroenterology and Hepatology*.

[20] Hoover J. G., Schumaker A. L., Franklin K. J. (2009). Use of intravenous proton-pump inhibitors in a teaching hospital practice. *Digestive Diseases and Sciences*.

[21] White K. T., Storch I. M., Mullin G. E. (2003). Intravenous pantoprazole: physician understanding of indications and their prescribing patterns leading to hospital restrictions. *The American Journal of Gastroenterology*.

[22] Lang E. S. (2008). Intravenous proton pump inhibitors prior to endoscopy in suspected upper gastrointestinal bleeding. *Canadian Journal of Emergency Medicine*.

[23] Sreedharan A., Martin J., Leontiadis G. I. (2010). Proton pump inhibitor treatment initiated prior to endoscopic diagnosis in upper gastrointestinal bleeding. *Cochrane Database of Systematic Reviews*.

[24] Craig D. G. N., Thimappa R., Anand V., Sebastian S. (2010). Inappropriate utilization of intravenous proton pump inhibitors in hospital practice—a prospective study of the extent of the problem and predictive factors. *An International Journal of Medicine*.

[25] Afif W., Alsulaiman R., Martel M., Barkun A. N. (2007). Predictors of inappropriate utilization of intravenous proton pump inhibitors. *Alimentary Pharmacology and Therapeutics*.

[26] Law J. K., Andrews C. N., Enns R. (2009). Intravenous proton pump inhibition utilization and prescribing patterns escalation: a comparison between early and current trends in use. *Gastrointestinal Endoscopy*.

[27] Shivkar Y. M. (2009). Clinical information in drug package inserts in India. *Journal of Postgraduate Medicine*.

[28] Khara H. S., Pitchumoni C. S. (2009). Proton pump inhibitors: a better prescription is needed. *Journal of Clinical Gastroenterology*.

[29] Hussain S., Stefan M., Visintainer P., Rothberg M. (2010). Why do physicians prescribe stress ulcer prophylaxis to general medicine patients?. *Southern Medical Journal*.

